# An Examination of Electronic Cigarette Content on Social Media: Analysis of E-Cigarette Flavor Content on Reddit

**DOI:** 10.3390/ijerph121114916

**Published:** 2015-11-20

**Authors:** Lei Wang, Yongcheng Zhan, Qiudan Li, Daniel D. Zeng, Scott J. Leischow, Janet Okamoto

**Affiliations:** 1 College of Management and Economics, Tianjin University, Tianjin 300072, China; E-Mail: l.wang@ia.ac.cn; 2 Department of Management Information Systems, University of Arizona, Tucson, AZ 85721, USA; E-Mails: yongchengzhan@email.arizona.edu (Y.Z.); zeng@email.arizona.edu (D.D.Z.); 3 The State Key Laboratory of Management and Control for Complex Systems, Institute of Automation, Chinese Academy of Sciences, Beijing 100190, China; 4 Mayo Clinic, Scottsdale, AZ 85259, USA; E-Mails: Leischow.Scott@mayo.edu (S.J.L.); Okamoto.Janet@mayo.edu (J.O.)

**Keywords:** e-cigarette flavor, social media, Reddit, e-juice, e-liquid

## Abstract

In recent years, the emerging electronic cigarette (e-cigarette) marketplace has shown great development prospects all over the world. Reddit, one of the most popular forums in the world, has a very large user group and thus great influence. This study aims to gain a systematic understanding of e-cigarette flavors based on data collected from Reddit. Flavor popularity, mixing, characteristics, trends, and brands are analyzed. Fruit flavors were mentioned the most (*n* = 15,720) among all the posts and were among the most popular flavors (*n* = 2902) used in mixed blends. Strawberry and vanilla flavors were the most popular for e-juice mixing. The number of posts discussing e-cigarette flavors has increased sharply since 2014. Mt. Baker Vapor and Hangen were the most popular brands discussed among users. Information posted on Reddit about e-cigarette flavors reflected consumers’ interest in a variety of flavors. Our findings suggest that Reddit could be used for data mining and analysis of e-cigarette-related content. Understanding how e-cigarette consumers’ view and utilize flavors within their vaping experience and how producers and marketers use social media to promote flavors and sell products could provide valuable information for regulatory decision-makers.

## 1. Introduction

In recent years, the emerging e-cigarette marketplace shows great development prospects all over the world. According to data from Euromonitor International, the global sales of vaping devices will hit £6 billion in 2015, as business in its largest market, the US, will more than double to £1.7 billion [1]. With tremendous growth in the e-cigarette marketplace, there have been heated discussions as to regulatory approaches to e-cigarettes, prompting significant research interest and regulatory concern.

The consumer experience of e-cigarettes depends on various factors, such as the structure of the cartridge or tank, the material of the mouthpiece, and the liquid of the solution. The solution, which is called e-liquid, or e-juice, is differentiated by concentration of nicotine, price, safety, and flavor. A better and smoother taste may increase enjoyment of the vaping experience.

Flavor has been found to be an attractive factor to e-cigarette adopters [2,3,4]. Fruit flavors have been found to be the most popular among users to date, while tobacco flavors have been more important for initiation of e-cigarette use among current smokers [5]. For traditional combustible cigarettes, menthol has been found to be most popular among African-Americans and younger consumers and is the only flavor of traditional cigarettes not banned by the FDA in the United States [6].

Tobacco companies have already successfully marketed traditional tobacco products to youth by using flavor varieties. For example, Kostygina *et al.* found that menthol and candy-like flavors increased little cigars’ and cigarillos’ appeal to starters by masking the heavy cigar taste [7]. This leads to the FDA banning or limiting such practices as they were deemed to specifically target minors. Similarly, the FDA has expressed concerns that flavored e-cigarettes could attract youth and lead them to take up smoking and become susceptible to the diseases and premature deaths it causes [8].

Vapor stores, stores selling e-cigarette devices and liquids, have relied heavily on the internet and social media for marketing and promotion, preferring its more interactive and immediate format and low cost to target current and potential users. Flavor is broadly used, both in online social media advertisements and offline store promotions, to increase the appeal of e-cigarette products [9]. Thus, the study of e-cigarette flavors, and in particular how social media is used to promote flavors and in turn e-cigarette products, is of great significance to regulatory decision-makers and public health advocates in order to better understand the use and initiation of e-cigarettes.

Although e-cigarette research literature is growing steadily, there are still significant gaps. For consumers, e-cigarette vendors, healthcare providers, and policy makers, a better understanding of the characteristics of e-cigarette flavors could serve to inform the current debate around the use, initiation, and short- and long-term effects of e-cigarettes. E-liquid is an important factor of e-cigarette usage. For example, Lerner examined the reactive oxygen species (ROS) associated with e-cigarette components and the aerosols that were inhaled by the user. They found that some constituents with oxidizing properties associated with e-cigarettes were health hazards that warrant further examination [10]. Jensen *et al.* studied the formaldehyde produced by vaping e-juice. They found that long-term vaping is associated with an incremental lifetime cancer risk of 4.2 × 10^−3^, assuming that inhaling formaldehyde-releasing agents carries the same risk per unit of formaldehyde as the risk associated with inhaling gaseous formaldehyde. This risk is five times higher than smoking one pack of conventional tobacco [11]. Both papers mentioned that flavors were an important component in e-juice. However, very few studies have focused on this research topic.

Fuoco *et al.* found that e-cigarette liquid flavors cannot be considered a major influence parameter in particle concentration emission [12]. However, Behar *et al.* tested eight cinnamon-flavored e-juices and found these fluids could produce Cinnamaldehyde (CAD) and 2-methoxycinnamaldehyde (2MOCA), which were highly cytotoxic. Thus, the cinnamon-flavored e-juice could adversely affect e-cigarette users [13]. They also noted that most studies mention e-liquid flavor only briefly and as one possible factor that could increase the appeal of e-cigarettes [4,14,15,16,17]. Similarly, Bahl *et al.* found e-cigarette refill fluids were cytotoxic to human embryonic stem cell and mouse neural stem cell not due to nicotine but related to chemicals used to flavor fluids, such as Cinnamon Ceylon [18]. Farsalinos *et al.* evaluated sweet-flavored e-liquid for avoidable risk induced by presence of diacetyl and acetyl propionyl [19]. Lisko *et al.* measured 10 additive flavor compounds among 36 e-cigarette products, and found that added menthol might reduce harshness or more closely simulate the sensory experience of smoking traditional cigarettes [20]. Tierney *et al.* revealed 13 out of 30 products were more than 1% by weight flavor chemicals [21]. Research on e-liquid flavors has not been sufficient and many questions still remain. It is important to determine the most popular e-cigarette product flavors and the reasons why consumers choose one flavor over another. Analysis of the effects and influences of flavor popularity on product use and initiation is also critical to better understand the role that flavors play in the vaping experience. Finally, these insights are necessary in order to inform the public and policy makers for further discussion and debate.

Social media such as YouTube and Facebook have recently become a significant platform for health surveillance [22] and social intelligence [23]. For example, YouTube has become an important data source for consumers, and previous research has examined the information shared on YouTube related to smoking [24], smoking cessation [25], smoking imagery associated with cigarettes [26], smokeless tobacco [27], and little cigars [28]. One recent study on YouTube also found that flavor has been used as a key promotional element for the sale of e-cigarettes and related products [17]. Some videos claim that consumers should choose from the multiple flavors, such as chocolate and strawberry, to make them more attractive and appealing [17]. It was also found that the vast majority of information on YouTube about e-cigarettes promoted their use and depicted the use of e-cigarettes as socially acceptable [17]. An analysis of 365 e-cigarette-related videos found that they highlighted e-cigarettes’ economic and social benefits, featuring a low level of fear appeal and negative message valence and a high level of marketing information [8]. Hua *et al.* used YouTube videos to study users’ puff duration and found it was approximately twice as long as puff duration for conventional smokers [29]. In another paper, Hua *et al.* used data collected from three forums, Electronic Cigarette Forum, Vapers Forum, and Vapor Talk, to study the symptoms caused by e-cigarettes. The symptoms were classified into medical categories with positive or negative effect and the association between symptoms was examined [30]. Research on other platforms found that Twitter appeared to be an important marketing platform for e-cigarettes [31]. Another well-known social media platform, Facebook, was studied by Liang *et al.* Their research primarily focused on the social networks constructed from fan pages [32]. In general, Twitter research focused on detection of trends and patterns. YouTube provides rich information in videos; thus the research in YouTube was more practical and specific. However, Reddit, an important social media platform, has not been thoroughly studied. Therefore, the current study examined data from Reddit and conducted content analysis based on information shared on this platform.

Reddit, founded in 2005, is essentially an online bulletin board system that provides news, entertainment, and social networking functionality. Users provide all the content and decide, through voting, the ranking of posts and comments [33]. As one of the most popular forums in the world, Reddit has great influence and a huge number of user groups. Reddit users are anonymous. Demographic information was not required when a user signed up. Even email was optional in registration. The loose requirement creates a highly free environment for users to discuss and communicate with each other. However, it also creates the difficulty of defining characteristics of users. As of 28 June 2015, Reddit had 163,966,958 unique visitors hailing from over 212 different countries, viewing a total of 7,086,828,967 pages [34]. Since 2008, Reddit allows users to create communities (called “subreddits”) where they can discuss interesting topics. Some research about health has been done based on the data collected from Reddit. Pavalanathan and Choudhury used Reddit to study mental health [35]. Arthur used Reddit to track the 2014 Ebola outbreak [36]. An article from the Institute for Health Research and Policy at the University of Illinois at Chicago pointed out that there was rich conversation happening in subreddits dedicated to electronic cigarettes and quitting smoking [37]. There are many publicly available posts about e-cigarettes and flavors, which have the potential to influence e-cigarette and flavor-related attitudes, choices, and behaviors. We believe the discussion of e-cigarette flavors in Reddit could profoundly influence readers subscribing to this community.

Despite the growing amount of literature on e-cigarettes on YouTube, Twitter, and Facebook, there are no published studies to date that have systematically mined e-cigarette and flavor content on Reddit. Given the potential that Reddit has to promote e-cigarette use through user-generated content or covert advertising, this study aims to gain a systematic understanding of the characteristics of a variety of flavors, popular flavors, and the reasons why they are popular by analyzing e-cigarette flavor-related posts and user information on Reddit.

## 2. Methods

### 2.1. Data Collection

E-cigarette flavor-related posts were collected from Reddit from 1 January 2011 to 30 June 2015 for study purposes in line with methods from several previous and related studies on Facebook and YouTube [17,32]. In these studies, a wide range of data was collected based on several keywords related to e-cigarettes. Some additional rules were applied to make sure that data was accurate and relevant. The data coding processes to classify the records were conducted manually. We used a similar approach. From previous studies [17,31], we identified the following seven keywords for data collection: electronic cigarettes, e-cigarettes, ecigarettes, e-cigs, flavor, flavors, and e-juice. Several subreddits were returned from this initial search. We chose the top 10 popular and relevant subreddits in ranking: /r/electronic_cigarette, /r/ecigclassifieds, /r/ejuice, /r/Vaping101, /r/ejuice_reviews, /r/EJuicePorn, /r/DIY_eJuice, /r/ecig_vendors, /r/Vaping, /r/E_cigarette. We believe that 10 subreddits could provide enough posts and comments for data analysis, and at the same time, a comprehensive understanding could be gained from analyzing a range of subreddits rather than a specific subreddit.

Two strategies were used to pull posts from the identified subreddits: (a) keyword searches and (b) ranking by relevance, hot spot, importance, up-to-date information, and reply count. Hot spot was given by Reddit search engine, and calculated by comprehensively considering of the number of browsing, comments, upvotes, and downvotes of a specific post. These strategies were chosen to mimic typical user behavior. Using these strategies, our dataset contained 493,994 posts on Reddit.

In practice, there is some noise in the posts due to semantic ambiguity. For example, the word “apple” not only refers to apple flavor, but also has different meanings in other contexts. For instance, “Snapple Apple” is a kind of apple beverage, while “apple watch” is an electronic product produced by the Apple Company. We consider words that are not relevant to e-cigarette flavors (e.g., “Snapple Apple”, “apple watch”) as noise. Thus, we eliminated posts with such noise. Finally, a total of 27,638 unique e-cigarette flavor-related posts and 7376 brand-related posts were identified for analysis.

### 2.2. Data Analysis

To gain a systematic understanding of the characteristics of a variety of flavors, popular flavors, and the reasons why they are popular, the following processes were carried out. First, two reviewers reviewed 2200 sample posts and classified the flavors based on their ingredients. In total, 29 flavors were identified into eight categories: fruit, cream, tobacco, menthol, beverages, sweet, seasonings, and nuts. We ran another test to classify these 29 flavors, and the kappa coefficient of this classification was 0.73. Based on the concept provided by Fleiss *et al.* [38], this kappa coefficient indicated substantial or good agreement.

Second, the number of times that each flavor occurred in posts was counted. Using this method, popular flavors can be identified and flavor categories created and/or confirmed. Furthermore, the evolution trend of each flavor category over time can be analyzed.

Third, mixed flavor patterns were analyzed. Searches for mixed flavor posts were conducted using the following search keywords: mix, mixes, mixed, premixed, blend, blended, and blends. The most popular two-flavor and three-flavor combinations were determined.

Finally, by analyzing brand-related posts, the top 10 most popular e-liquid brands were identified. Characteristics of a variety of flavors were also identified in order to attempt to gain a better understanding of the reasons why some flavors are more popular than others.

## 3. Results

### 3.1. Classification of Flavors

E-cigarette flavor-related posts were manually reviewed and classified into eight categories: Fruit, cream, tobacco, menthol, beverages, sweet, seasonings, and nuts. As shown in Table 1, these eight flavor categories include 29 different flavors identified in Reddit posts.

Previous research used a questionnaire to survey e-cigarette consumers and identified seven categories: tobacco, mint/menthol, sweet, nuts, fruit, drinks/beverages, and other [5]. In addition to these, we further differentiated some categories. Cream flavors were differentiated from sweet ones as some flavors were not only characterized as sweet, but also as smooth and creamy. For example, cream flavors included chocolate, vanilla, and milk as compared to honey and candy for sweet flavors. This research is the first to identify the seasonings category, which are interesting flavors derived primarily from various spices.

**Table 1:** Characteristics of flavors.

Categories	Characteristics	Users’ Posts
Fruit	Fruit category flavors taste sweet, do not stimulate, are mild, and are closest to the naturally occurring flavor of fruit	Strawberry is excellent with dark and murky flavors such as tobaccos, chocolates, hazelnuts. Not just fruit alone, but the balance is delicate and takes some work. I got the chocolate banana, it improves a bit after steeping a week. I agree with what you say on the cherry it was the best, but a little sickening. I also got strawberry cream, it was ok but kind of had a strange aftertaste from the cream part. The banana doesn’t taste as candied as other banana liquids I’ve tried, which is a redeeming quality for me. However, the banana flavor is a bit more pronounced than I would have hoped for, with the other flavors taking a back seat. This juice seems much sweeter than Baked Blue. I’m experiencing almost the same level of harshness here. A sweet natural cherry e-juice that will definitely take you back to that Greek bar a few summers ago that served delicious cherries on ice. I won’t put it in that many words but if you ever kissed a girl (and liked it!) with cherry lipbalm you’ll know how this tastes immediately. It’s a sweet and candylike cherry flavour with a nice touch. I quite like it, it reminds me of my first kiss. (aww)
Cream	Cream category flavors are sweet and smooth. The exhale leaves the creamy taste.	All of the chocolates are ok though not massively inspiring, the coffees are very chemical and cinnamon is so bloody strong it’s insane. More bakery goodness here. Sweet, creamy, with a good full cookie note. Not an all day vape for me, it’s a bit rich, but it’s a flavour I’m happy to spend an evening with sometimes. Vanilla: Decent, there are better vanilla flavours available, but also worse.
Tobacco	Not all tobacco category flavors taste the same. In general, some tobacco flavors are used to mimic the real cigarette flavors to help people switch from real cigarettes to e-cigarettes. Other tobacco flavors are blended with sweeteners to have sweet, smooth, and fragrant taste. These kind of flavors are more appealing to e-cigarette users who are not interested in the traditional tobacco taste.	I like only the standard tobacco flavored juice. It took me a little bit to acquire the taste for it, but I prefer it over an analog at this point. I have tried several of flavors though. Things like blueberry, cherry, apple, menthol *etc*. The blueberry was ok, the rest were not very good. But the blueberry tasted like grape kind of so it was weird. I have tried the flavoring specific to brands, like Camel *etc*, and they all taste like regular flavor with a cherry aftertaste to me. Weird but that is what myself and my friends taste each time. So I stick to the standard kind, which tastes nothing like tobacco to me, but is the best flavor for a smoker. It’s a clove tobacco. And quite a bit different from what I expected. Clove is prominent but not overpowering. Tobacco is there, but not overly so. Reminds me of burley pipe tobacco. Mild but pleasant. In my opinion tobacco flavors don’t have enough sharp edges and need a little bite, but this might be solved if you like a higher PG level in your juice. If throat hit isn’t important to you, all the better.
Menthol	Menthol category flavors are slightly bitter on the inhale, offer a light throat hit, and have a cooling sensation in the nasal cavity and on the exhale. Some people reported there is/it has a harsh taste.	The fresh breath is a minty menthol kind of flavour. It doesn’t actually freshen breath but tastes like it. I remember when candy-flavored cigarettes were banned. My sister (teen at the time) tried them and found them awful. “If I wanted candy, I’d just eat candy. If I want to mask the flavor of a cigarette, I’ll smoke a menthol.” Menthol cigarettes are most commonly smoked by kids 18 and under (in my experience, anyways). Yet we ban clove cigarettes because they’re trying to lure in kids, but menthol is still cool... New to vaping, stopped smoking a few months ago, didn’t care much for e cigs, but a friend gave me a vape set up and I’m going to try and keep using it. I don’t care much for any fruity flavors, not a fan. I want to stay away from tobacco flavors to reduce my urge to smoke an actual cigarette. I tried a (Red Rock Vapor) mint blend, and it was horrid. Way too harsh and peppermintish. I liked (LA vape) bubblegum mixed with a hint of (Pure) cinnamon, any other suggestions to mix bubblegum with? I have some (Pure) cappuccino that I mixed with (Red Rock Vapor) vanilla which I really enjoyed as well.
Beverages	Beverages category flavors have a rich aroma, a smooth inhale, and a nice thick vapor production.	I had a dream. A dream that this coffee flavoring would open up doors for me. That I could be the new Grant’s Vanilla Custard. For those who don’t know what I’m talking about, I found this flavoring meant for beverages at my workplace and it had the same ingredients as e-juice (without the nicotine.) So I decided to give it a whirl and vape it. I bought Green Tea E-Liquid with 15 mg nicotine and PG 60%, VG 20% and 20% flavorings. At least that’s what’s written on the bottle. I gave it to my mom for a spin (30+ year smoker) and she likes it and wants me to buy one for her as well. But after browsing reddit and a few forums, I’m starting to wonder if there are better options than the airsmoke one, because it’s the only one available in my country. My question basically is—do you know good companies that ship to Eastern Europe (Latvia) and what would you recommend for me and my mother. So far my top flavors from Five Pawns have been Absolute Pin and Perpetual Check. I would like to find a Chai Tea flavor, I think that would be good.
Sweet	Sweet category flavors taste sweet and mild, but they do not produce too much vapor.	I hate VG—Anytime I go over 50/50 I can taste that disgusting sweet VG flavor that tends to kill the actual flavor of the juice. I’m not a big fan to sweet flavors—If you didn’t guess from my hate of VG. I like multiple tastes—I love Five Pawns for the layers that they have in their juice, it’s not just peaches and cream which comes out as a mono flavor. I don’t like mint. Cotton candy 6 mg. Surprising. I wasn’t expecting much from this flavour, but had seen several posts about how good it can be. It’s definitely up there with other smooth, sweet flavours. Not perfumey, just candy goodness. The candy flavors are the reason I was able to quit smoking. They helped me realize ... I hate the way menthol cigs, and even clove cigs taste.
Seasonings	Seasonings’ category flavors are really unique and usually mixed with other flavors.	The cinnamon is very much like the dark gooey stuff in a cinnamon roll. There’s a good note of icing to it, and of course a full bakery note as well. It’s wonderful. So the idea is to concoct a tobac/black pepper/leather flavored juice, a layered flavor that hits the palate with black pepper and tobac, then eases into something vaguely like “that new leather/new car aroma”, all gently fading into a long sweet birch finish. I have the pepper and a good tobacco flavoring ... long sought out for their ability to stand apart distinctly and layer in concert. I currently have Cinnamon roll from mtbakervapor, 18 mg nic. 80% VG 20% PG, 4 extra flavor shots. It has an amazing flavor long-term, as this is my second bottle. It also gives these wonderful clouds of smoke, and hits pretty smooth.
Nuts	Nuts category flavors are mild and can be mixed with other flavors.	I have an allergy to nuts in general, primarily peanuts (on the allergen blood test peanuts were given the second highest rating—Anaphylaxis shock after consumption). I carry an epipen at all times just in case of accidental exposure. I have personally made juice with a variety of different nut/peanut butter flavors and I have never had an issue vaping them or with accidental contact with my skin. Granted, the smell of TFA peanut butter still makes me nauseous due to the association I have obviously developed between the smell of peanut butter and potential death haha. Om Nom is a reference to a local flavor. It’s a banana nut bread and cinnamon flavor. The banana nut was really strong on the exhale, with a hint of cinnamon. So I’m new to making e juice and I’m definitely more of a cloud chaser so I tend to be higher with vg maybe like a 80 vg 20 pg and most of the juices I buy are that ratio. My juices are so dull. The flavors are extremely muted and I’m following a calculator to do the mixes. Some have been very good at first but then the next day they are muted. I’m familiar with vapors tongue but I mean these are really muted. A good example was today I made a 7% banana cream 10% peanut butter both lorann flavoring. Any tips or advice anyone can give me?

### 3.2. Flavor Characteristics

Many users shared their experiences of using e-cigarettes with different flavors on Reddit. From the data set we have collected, the active users consist of e-cigarette vapers, reviewers, vendors, individual e-juice makers, *etc*. Different users have different perspectives of these flavors. We collected some characteristics of these flavors described by the users, which are shown in Table 1.

In the table above, we selected three posts for each category of flavors and summarized some characteristics. Fruit flavors taste sweet, do not stimulate, are mild, and are closest to the naturally occurring flavor of fruit. User 1 provided some mixing patterns for fruits flavors. User 2 expressed the feeling that banana flavor was “harsh”. User 3 described the taste of cherry flavor as the feeling of “kissing a girl”.

Cream category flavors are sweet and smooth. The exhale leaves the creamy taste. User 1 talked about mixing chocolate flavor. User 2 preferred cream flavor User 3 reviewed e-juice, including a comment about a vanilla flavor from a specific brand.

Tobacco flavor is more complicated. Some tobacco e-juices are used to mimic the conventional cigarette. However, others are blended with sweeteners to appeal to e-cigarette users who are not interested in the traditional tobacco taste. User 1 insisted the classical tobacco flavor was the best flavor for a smoker. However, user 2 was fond of a milder tobacco taste, and user 3 discussed approaches to increase the throat hit when using tobacco flavors.

Menthol category flavors are slightly bitter on the inhale, offer a light throat hit, and have a cooling sensation in the nasal cavity and on the exhale. Some people report that it tastes harsh. User 1 compared the taste of minty menthol with fresh breath. User 2 mentioned that the menthol flavor could be used to mask the traditional tobacco taste. User 3 was a new user and went to Reddit asking for some suggestions.

Beverages category flavors have a rich aroma, a smooth inhale, and a thick vapor production. User 1 expressed their enjoyment of using coffee flavor. User 2 talked about how his/her mother loved the tea flavor and asked for some information about e-cigarette vendors in Latvia. User 3 said he/she would like to find a Chai tea flavor.

Sweet category flavors are sugary but not greasy as cream. User 1 just did not like the sweet flavors because they did not like the taste of VG. User 2 loved the cotton candy flavor very much, and user 3 said the candy flavor could be helpful in quitting smoking.

Seasonings category flavors are unique and usually are mixed with other flavors. User 1 said that the cinnamon flavor was similar to the taste in a cinnamon roll, which was “wonderful”. User 2 discussed the pepper flavor. User 3 shared the composition of a kind of cinnamon e-juice from Mt baker vapor.

Nuts category flavors are mild and can be mixed with other flavors. User 1 was allergic to nuts but still made DIY e-juice with nuts flavors. However, this user had never had an issue vaping them or with accidental contact with skin. Granted, the smell of TFA peanut butter still produced a nauseous feeling due to the association they had developed between the smell of peanut butter and an allergic reaction. User 2 seemed to be a local e-juice vender promoting its products. User 3 discussed experience with making DIY e-juice and asked for help.

In general, the user experiences with these different flavors were different. Their evaluation covers a wide scope. There was plenty of discussion of mixing blends, which seems to be a common trend.

### 3.3. Popularity Analysis of Flavors

#### 3.3.1. Single Flavor

The number of times that each flavor category and flavor word occurred in posts was counted. If a post mentioned several flavors, all the flavors mentioned would be counted once. If a post contained apple three times and banana one time, we count the number of posts that contained apple as one, and the number of posts that contained banana as one. Table 2 shows the breakdown of posts for each flavor category and specific flavor. We identified a total of 27,638 unique e-cigarette flavor-related posts. The total number of flavors frequencies is 45,130. Thus the average of flavors per post is 1.63.

**Table 2:** Flavor category frequencies.

Category	Number of Posts	Flavor	Number of Posts
Fruit	15,720	Strawberry	3657
		Banana	1864
		Apple	1715
		Peach	1205
		Blueberry	1216
		Mango	1170
		Cherry	1030
		Orange	1007
		Lemon	922
		Watermelon	916
		Raspberry	730
		Pomegranate	288
Cream	10,289	Vanilla	3036
		Custard	2664
		Milk	1949
		Chocolate	1329
		Cake	810
		Cookie	501
Tobacco	7475	Tobacco	7475
Menthol	3421	Menthol	2322
		Mint	1099
Beverages	3347	Coffee	1876
		Tea	1205
		Wine	266
Sweet	2711	Candy	1612
		Honey	1099
Seasonings	1415	Cinnamon	1271
		Pepper	184
Nuts	752	Nuts	752

Fruit, cream, and tobacco were the most popular flavor categories, while nuts and seasonings flavors were the least mentioned. It should be noted, however, that many posts discussed several different flavors in the same post. When multiple flavors were mentioned in a single post, it was most often:

(1) A comparison of several flavors
(2) An inquiry for introduction of different flavors
(3) A demonstration of mixing flavors
(4) A promotion of different flavors

Many users shared their e-juice DIY experience. They believed DIY flavors could produce better taste. However, there is a concern that mixing different flavors created some additional risk. Such combinations might produce chemicals harmful to e-cigarette users. Therefore we think monitoring the mixing of flavors is important. We hope the patterns detected in this research will be further examined from a public health perspective. The next section contains further analysis on mixing flavors.

#### 3.3.2. Flavor Mixing/Combination Analysis

**Figure 1:**
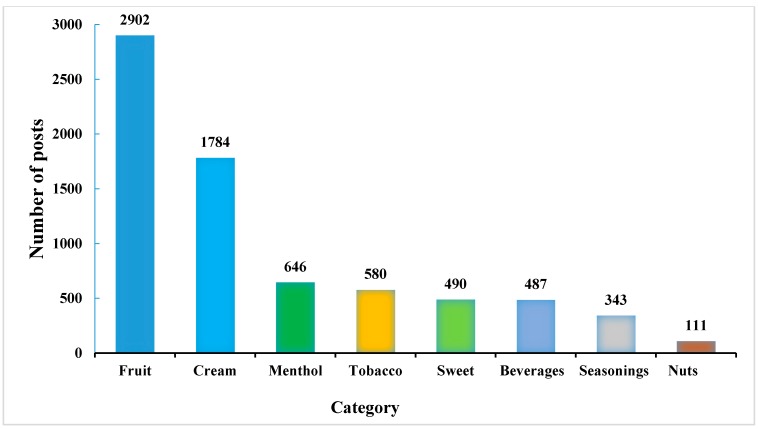
Flavor frequencies from multi-flavor posts.

Many users discuss mixing different flavors together to create new flavor combinations. The 2885 posts that mentioned multiple flavors were further analyzed to examine patterns of mixed/combined flavors. Figure 1 presents the number of posts that mentioned the type of flavor. The results of this analysis show that flavors in fruit and cream categories are most often used to mix with other flavors.

**Figure 2:**
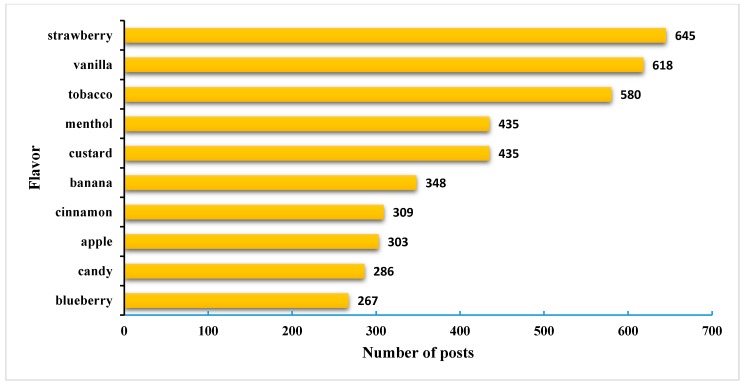
Most popular flavors in multi-flavor posts.

However, if single flavors only are examined rather than flavor categories, as shown in Figure 2, strawberry is the most popular flavor used in mixed e-liquids, followed by vanilla and tobacco flavors. The top 10 flavors include four fruit flavors and two cream flavors, which is consistent with the flavor category findings above.

The most popular combinations of two- and three-flavors were also examined. The posts indicate that combinations of vanilla and strawberry flavors are the most popular among the Reddit users (Table 3 and Table 4).

**Table 3:** Most popular two-flavor combinations.

Ranking	Flavor Combination	Number of Posts
1	custard + vanilla	259
2	strawberry + vanilla	202
3	strawberry + banana	151
4	tobacco + vanilla	150
5	custard + strawberry	136

**Table 4:** Most popular three-flavor combinations.

Ranking	Flavor Combination	Number of Posts
1	custard + vanilla + strawberry	99
2	strawberry + vanilla + banana	70
3	strawberry + vanilla + apple	65
4	custard + vanilla + tobacco	61
5	custard + vanilla + cinnamon	61

### 3.4. Temporal Trend Analysis of Flavor Categories

**Figure 3:**
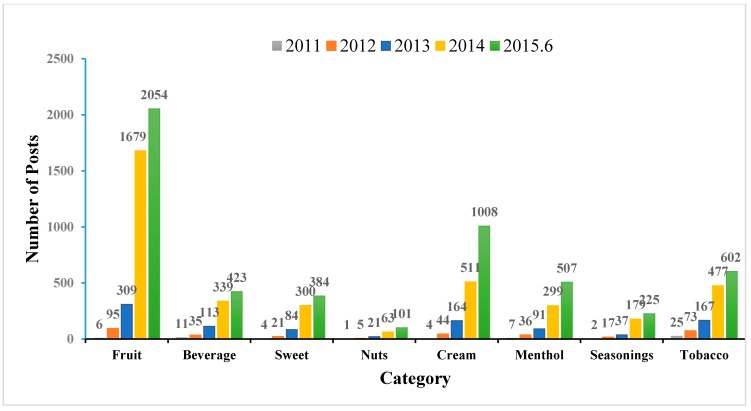
Temporal trends for flavor categories.

Since 2014, many U.S. tobacco companies have entered the e-cigarette market, triggering rapid growth and expansion. It can be seen in Figure 3 that the number of discussions in each flavor category increased significantly beginning in 2014. According to this growth trend, the number will continue to increase through 2015. The fruit category is clearly the most popular on Reddit, with the post volume regarding this flavor category rising the most sharply since 2014. Similarly, other flavor categories, such as cream and sweet, also show an increased discussion post volume due to the fast-developing market. Here, we only examined the increase on category level. However, a flavor-level analysis could provide detailed information on trends for a specific flavor.

### 3.5. Popular Flavor Brand Analysis

**Figure 4:**
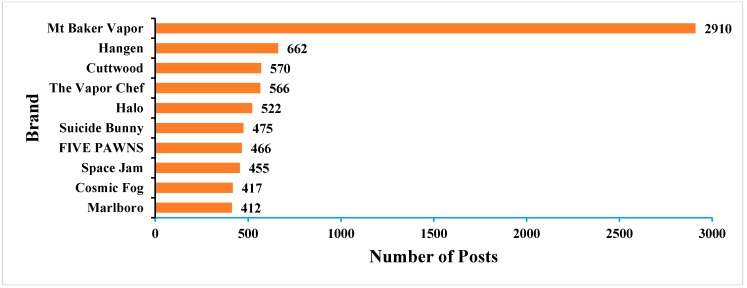
Top-10 brands.

Although health benefits and possible adverse effects of e-cigarettes are still unclear and need further study by the scientific community, many smokers show great interest in information regarding the safety and quality of e-liquids used with e-cigarettes. Well-known and/or well-advertised brands, such as Mt Baker Vapor and Hangen, receive good reviews from consumers and are popular among users who report good quality and taste. The top 10 most popular e-liquid brands are shown in Figure 4.

Among these brands, Mt Baker Vapor, Hangen, Halo, and Marlboro produce both e-juice and e-cigarette devices. All the other brands only sell e-juice. The characteristics of these brands showed that Reddit was a good promotion platform both for big names in the industry and small companies with high-quality products. The comprehensive composition of users might increase the promotion power of this platform.

## 4. Discussion

To the best of our knowledge, this is the first systematic study of e-liquid flavors based on data collected from social media. Popularity and characteristics of flavors are summarized based on data collected from Reddit, a social media platform that also has not been studied in the context of e-cigarette content. In this research, the popularity of flavors was the primary focus, which was defined as the number of posts containing the flavor. Although preference and sentiments were not analyzed in this study, we believe the number of posts could be considered as a good index for the prevalence of flavor use. The main findings are as follows:

(1) Through analyzing e-cigarette flavor-related posts, 29 different flavors were classified into eight categories, and the fruit category was the most popular flavor category, which is consistent with previous studies [5]. In addition, sweet category flavors and cream category flavors were also very popular. Smaller numbers of users were interested in the tobacco and menthol categories.
(2) We find that the fruit and cream flavor categories are most often mixed with other flavors. Strawberry is the most popular flavor used in mixing blends. Finally, vanilla and strawberry are mentioned most in multi-flavor combinations by Reddit users.
(3) The results of temporal trend analysis show that the number of posts in each flavor category has grown steadily since 2011, which correlates with the sharply growing e-cigarette market. As of 2014, there have been four generations of devices [39], and sales of e-cigarettes are estimated to grow 24.2% per year through 2018 [40]. Our data collected from Reddit appear consistent with this noted growth.

Based on the findings above, the fruit category is the most popular flavor among e-cigarette users. The reason might be that fruit flavors taste sweet, are mild, do not stimulate, and are closest to the actual naturally occurring flavor of fruit, since they have a relatively high glycerol ratio. This could potentially contribute to use and uptake of e-cigarette smoking by making products and the vaping experience more appealing. The large variety and diversity of fruit flavors could also be a reason they are the most popular and appeal to the most users. Research on little cigars and cigarillos has found that candy-like flavors could increase the appeal to starters because it masks the heavy cigar taste [7]. Similarly, adding candy-like and other non-tobacco flavors could potentially be perceived as enjoyable, which might make it easier for e-cigarette companies to recruit new users.

The prevalence of flavors in e-cigarettes raises new concerns about this product, which is advertised as an approach to help smokers quit. However, the large variety of sweet flavors could also increase appeal to adolescents and young adults, which could be supported by evidence similar to that used by the FDA to ban the use of flavors (with the exception of menthol) in traditional combustible cigarettes. Flavor use in e-cigarettes, which is not currently banned, has been proposed by public health advocates as just the next in a long list of tactics used by producers and marketers to attract adolescents and young adults to initiate smoking behaviors. Our research suggests that, e-cigarette flavors, especially fruits flavors, are attractive to vapers. The health effects and the attractiveness of flavors should be further examined to help policy makers to determine what regulatory action is appropriate for flavored e-cigarettes. E-cigarette flavorings could potentially be harmful to humans if inhaled into the lungs. Prior research found that the concentrations of some flavor chemicals in e-cigarette fluids are sufficiently high for inhalation exposure by vaping to be of toxicological concern [21]. Another investigation found that flavoring is a parameter known to affect the stability of products. For example, nicotine was often easily oxidized by common substances found in mint, vanilla, and fruit flavors. The oxidative degradation of nicotine resulted in high amounts of nicotine-related impurities, which was harmful to human bodies. Furthermore, based on their sampling, some brands had levels of impurities above accepted limits for pharmaceutical products [3]. Our findings suggest that fruit flavors, in particular, should be the first to receive further investigation from the medical and public health communities.

## 5. Contributions, Limitations, and Future Research

### 5.1. Contributions

In summary, e-cigarette flavors could be a possible factor that contributes to smoking initiation. Our findings, based on Reddit e-cigarette-related content, reveals that fruit flavors are the most popular and that this flavor category should perhaps be the first to be investigated further in future research.

This study is also the first study to examine Reddit as a social media data source for e-cigarette research. The user-generated content on Reddit is likely to be different than on other social media sites. Further analysis and comparison of e-cigarette content across social media platforms is needed. This study and its findings based on Reddit should serve as the first example for data mining in this platform.

This is also the first research that we are aware of that investigates flavor combinations mixed by e-cigarette users. Rather than a single e-liquid flavor, consumers are increasingly mixing multiple flavors to create a more complex and unique vaping experience. We find that fruit and cream flavor categories are the most popular used in mixing. The mixing of different flavors could potentially change the substance and components in the e-liquid, creating new compounds for which even less is known regarding long-term health effects. We hope our findings in this study could be analyzed by toxicological experiments to find out the potential health effects of flavor mixing.

### 5.2. Limitations

We collected data on Reddit from 1 January 2011 to 30 June 2015 to the extent feasible, but posts and comments beyond this scope were not collected. Including all the data could provide more comprehensive understanding of the patterns of e-cigarette flavors. However, we believe the current dataset of 493,994 posts is large enough for some data analysis and mining processes. In this research, we treated posts and comments as the same. Thus, relationships and network structures of the posts and comments were not included in the analysis, and interactions of users were omitted. This information could be used in more detailed description and prediction models.

As for the data collection strategy, we used keywords from current literature to identify e-cigarette-related posts: electronic cigarettes, e-cigarettes, ecigarettes, e-cigs, flavor, flavors, and e-juice. However, another important keyword we missed is vape or vaping, which is widely used among e-cigarette users to discuss the behavior of smoking e-cigarettes. We may have overlooked some valuable data because of this flaw. However, because of the huge amount of posts and comments, vape and vaping were also widely used in the posts we collected. Thus, we still believe the validity of our research findings. In future research, we would like to expand the keywords set for a more comprehensive dataset.

We were interested in the Reddit user characteristics, however, age and gender information were not available and hence could not help us define more important and interesting patterns on e-cigarette adoption.

Another limitation is the categorization of flavors. We only listed major flavors mentioned in the posts. Some other flavors such as sugar cane, apple vinegar, and absinthe are not included because they appeared infrequently. Although these flavors are not as common, additional analyses are needed to characterize all flavors because certain flavors might not be heavily used by large populations but could be used by specific populations, such as youth.

Finally, this study only focused on the prevalence of flavors, not the sentiments of posts and comments. The user preference of flavors is closely related to positive or negative content. For example, “I hate strawberry e-juice” and “I love strawberry e-juice” have totally different preference meaning. However, they all positively contribute to the popularity analysis we performed. Thus, this research determined popular or prevalent flavors, but not preferred flavors.

### 5.3. Future Research

We envision three possible approaches for further study. First, as stated above, certain flavors or flavor constituents could be hazardous to human health. Flavors are sold in stores and via the internet to a wide variety of users even though no medical testing of those flavors has been conducted, so the constituents of those flavors are unknown. The U.S. FDA has indicated that they intend to regulate e-cigarette products, and flavors, so future research will need to be conducted to determine health risks. We also examined flavor mixing/mixtures. Since cinnamon was found to contain highly cytotoxic substances [13], flavors blends including cinnamon should be tested. Cream is an interesting new category that could be further explored.

Future informatics analyses can also inform understanding of flavor and e-cigarette use, such as self-reported associations and relationships in social media between flavor and short-term discomfort and long-term symptoms. For example, our analysis of social media comments on Reddit found that some users reported xerostomia, eructation, and allergy after trying e-cigarettes. Some users expressed concern about the effect of sweet flavors on diabetes. Further exploration of how users describe the health effects of specific flavors might even function as an “early warning system” regarding potentially unsafe flavorants. Thus, further in-depth analysis of flavor characteristics and potential disease risk is a promising area of research.

Second, this study only focused on the prevalence of flavors, not the meanings of posts and comments. A more in-depth analysis of sentiments could reveal more information and patterns of flavors among e-cigarette users. For example, the discussion of an apple flavor could be considered as positive, negative, or neutral in sentiment, due to the difference in user-generated content. A broadly mentioned e-juice brand could be really great, or on the contrary, could be the worst. The two situations are the same in prevalence but different in sentiment. Thus, attending to the meanings of posts could reveal more information about e-cigarette users.

Finally, it is important to note that the flavors being discussed on Reddit do not provide information on the nicotine strength of the products. Different nicotine concentrations could potentially impact how different flavors are perceived or what reaction or health effects occur when they are consumed. For example, some users mentioned that tobacco flavor should be used in liquid containing high concentrations of nicotine because the “throat hit” would be more intense and enjoyable. Thus, further research could be conducted to analyze the matching and effect of flavors and nicotine solutions.

## 6. Conclusions

This is the first study showing that Reddit is heavily used by the e-cigarette and vaping community to share information about flavors and other aspects of e-cigarette use, and that Reddit social media data can be mined for valuable information on self-reported e-cigarette flavor use. We found that fruit flavors are the most popular among all the flavor categories, and postings indicate that combinations of vanilla and strawberry flavors are the most popular among Reddit users. This analysis of Reddit social media is an important step in understanding how and why consumers use different flavored e-cigarette products, in particular because Reddit data is far more difficult to analyze than some other social media venues (e.g., Twitter). Reddit data could function as an early warning system to better understand the emerging trends of e-juice flavors. In addition, future analyses of these data could lend insight into how consumers and e-cigarette businesses are reacting to new regulations and products. Consumers, e-cigarette producers, and policy makers could make use of this information to identify new products, health outcomes from particular products, and how products are being marketed/promoted, which could in turn inform the development and implementation of new regulations or laws.

## References

[1] Lauren Davidson Vaping Takes off as E-Cigarette Sales Break Through $6 bn. http://www.telegraph.co.uk/finance/newsbysector/retailandconsumer/11692435/Vaping-takes-off-as-e-cigarette-sales-break-through-6bn.html.

[2] Choi K., Fabian L., Mottey N., Corbett A., Forster J. (2012). Young adults’ favorable perceptions of snus, dissolvable tobacco products, and electronic cigarettes: Findings from a focus group study. Am. J. Public Health.

[3] Etter J.F., Zäther E., Svensson S. (2013). Analysis of refill liquids for electronic cigarettes. Addiction.

[4] McDonald E.A., Ling P.M. (2015). One of several “toys” for smoking: Young adult experiences with electronic cigarettes in New York City. Tob. Control.

[5] Farsalinos K.E., Romagna G., Tsiapras D., Kyrzopoulos S., Spyrou A., Voudris V. (2013). Impact of flavour variability on electronic cigarette use experience: An internet survey. Int. J. Environ. Res. Public Health.

[6] US Food and Drug Administration Overview of the Family Smoking Prevention and Tobacco Control Act. http://www.fda.gov/TobaccoProducts/GuidanceComplianceRegulatoryInformation/ucm246129.htm.

[7] Kostygina G., Glantz S.A., Ling P.M. (2014). Tobacco industry use of flavours to recruit new users of little cigars and cigarillos. Tob. Control.

[8] Paek H.-J., Kim S., Hove T., Huh J.Y. (2014). Reduced harm or another gateway to smoking? Source, message, and information characteristics of E-cigarette videos on YouTube. J. Health Commun..

[9] Cheney M., Gowin M., Wann T.F. (2015). Marketing practices of vapor store owners. Am. J. Public Health.

[10] Lerner C.A., Sundar I.K., Watson R.M., Elder A., Jones R., Done D., Kurtzman R., Ossip D.J., Robinson R., McIntosh S. (2015). Environmental health hazards of e-cigarettes and their components: Oxidants and copper in e-cigarette aerosols. Environ. Pollut..

[11] Jensen R.P., Luo W., Pankow J.F., Strongin R.M., Peyton D.H. (2015). Hidden formaldehyde in e-cigarette aerosols. N. Engl. J. Med..

[12] Fuoco F.C., Buonanno G., Stabile L., Vigo P. (2014). Influential parameters on particle concentration and size distribution in the mainstream of e-cigarettes. Environ. Pollut..

[13] Behar R.Z., Davis B., Wang Y., Bahl V., Lin S., Talbot P. (2014). Identification of toxicants in cinnamon-flavored electronic cigarette refill fluids. Toxicol. in Vitro.

[14] Pearson J.L., Richardson A., Niaura R.S., Vallone D.M., Abrams D.B. (2012). E-cigarette awareness, use, and harm perceptions in US adults. Am. J. Public Health.

[15] Cobb C., Ward K.D., Maziak W., Shihadeh A.L., Eissenberg T. (2010). Waterpipe tobacco smoking: An emerging health crisis in the United States. Am. J. Health Behav..

[16] Trtchounian A., Williams M., Talbot P. (2010). Conventional and electronic cigarettes (e-cigarettes) have different smoking characteristics. Nicotine Tob. Res..

[17] Luo C., Zheng X., Zeng D.D., Leischow S., Cui K., Zhang Z., He S. (2013). Portrayal of electronic cigarettes on YouTube. Smart Health.

[18] Bahl V., Lin S., Xu N., Davis B., Wang Y.H., Talbot P. (2012). Comparison of electronic cigarette refill fluid cytotoxicity using embryonic and adult models. Reprod. Toxicol..

[19] Farsalinos K.E., Kistler K.A., Gillman G., Voudris V. (2014). Evaluation of electronic cigarette liquids and aerosol for the presence of selected inhalation toxins. Nicotine Tob. Res..

[20] Lisko J.G., Tran H., Stanfill S.B., Blount B.C., Watson C.H. (2015). Chemical composition and evaluation of nicotine, tobacco alkaloids, pH, and selected flavors in e-cigarette cartridges and refill solutions. Nicotine Tob. Res..

[21] Tierney P.A., Karpinski C.D., Brown J.E., Luo W., Pankow J.F. (2015). Flavour chemicals in electronic cigarette fluids. Tob. Control.

[22] Yan P., Chen H., Zeng D. (2009). Syndromic surveillance systems. Annu. Rev. Inf. Sci. Technol..

[23] Wang F.-Y., Carley K.M., Zeng D., Mao W. (2007). Social computing: From social informatics to social intelligence. IEEE Intell. Syst..

[24] Freeman B., Chapman S. (2007). Is “YouTube” telling or selling you something? Tobacco content on the YouTube video-sharing website. Tob. Control.

[25] Backinger C.L., Pilsner A.M., Augustson E.M., Frydl A., Phillips T., Rowden J. (2011). YouTube as a source of quitting smoking information. Tob. Control.

[26] Forsyth S.R., Malone R.E. (2010). “I’ll be your cigarette-light me up and get on with it”: Examining smoking imagery on YouTube. Nicotine Tob. Res..

[27] Bromberg J.E., Augustson E.M., Backinger C.L. (2012). Portrayal of smokeless tobacco in YouTube videos. Nicotine Tob. Res..

[28] Richardson A., Vallone D.M. (2012). YouTube: A promotional vehicle for little cigars and cigarillos?. Tob. Control.

[29] Hua M., Yip H., Talbot P. (2011). Mining data on usage of electronic nicotine delivery systems (ENDS) from YouTube videos. Tob. Control.

[30] Hua M., Alfi M., Talbot P. (2013). Health-related effects reported by electronic cigarette users in online forums. J. Med. Internet Res..

[31] Huang J., Kornfield R., Szczypka G., Emery S.L. (2014). A cross-sectional examination of marketing of electronic cigarettes on Twitter. Tob. Control.

[32] Liang Y., Zheng X., Zeng D., Zhou X., Leischow S. (2013). An empirical analysis of social interaction on tobacco-oriented social networks. Smart Health.

[33] Frequently Asked Questions. https://www.reddit.com/wiki/faq#Whatdoesthenameredditmean.

[34] About Reddit. https://www.reddit.com/about.

[35] Pavalanathan U. (2015). Identity management and mental health discourse in social media identity in online communities. Int. World Wide Web Conf. Comm..

[36] Arthur M. (2014). Reddit: Tracking the 2014 Ebola outbreak across the world. Nurs. Stand..

[37] Buenger M. Shout-Out: Are You Using Reddit for Public Health Research? What Works?. http://www.healthmediacollaboratory.org/shout-out-are-you-using-reddit-for-public-health-research-what-works/.

[38] Fleiss J.L., Levin B., Paik M.C. (2003). Statistical methods for rates and proportions. Wiley Series in Probability and Statistics.

[39] Brandon T.H., Goniewicz M.L., Hanna N.H., Hatsukami D.K., Herbst R.S., Hobin J.A., Ostroff J.S., Shields P.G., Toll B.A., Tyne C.A. (2015). Electronic nicotine delivery systems: A policy statement from the American association for cancer research and the American society of clinical oncology. J. Clin. Oncol..

[40] Wahba P.U.S. E-Cigarette Sales Seen Rising 24.2% per Year through 2018. http://fortune.com/2014/06/10/e-cigarette-sales-rising/.

